# Increasing access to fertility preservation for women with breast cancer: protocol for a stepped-wedge cluster randomized trial in France

**DOI:** 10.1186/s12889-024-17719-3

**Published:** 2024-01-19

**Authors:** Maria Claudia Addamiano, Camille Joannes, Leslie Fonquerne, Charlotte Morel, Delphine Lauzeille, Lorène Belkadi, Fabienne Empereur, Pascale Grosclaude, Eric Bauvin, Cyrille Delpierre, Sébastien Lamy, Marie-Anne Durand

**Affiliations:** 1 EQUITY research team (Certified by the French League Against Cancer), CERPOP, UMR 1295, Université Toulouse III Paul Sabatier, Toulouse, France; 2 Regional Cancer Network of Occitanie (Onco-Occitanie), Toulouse, France; 3 Regional Cancer Network of Pays de la Loire (Onco-PL), Nantes, France; 4 Tarn Cancers Registry, Claudius Regaud Institute, Toulouse University Cancer Institute (IUCT- O), Toulouse, France; 5 The Dartmouth Institute for Health Policy & Clinical Practice, Dartmouth College, Lebanon, NH USA; 6 Unisanté, University Center for General Medicine and Public Health, Lausanne, Switzerland

**Keywords:** Fertility preservation, Breast cancer, Combined intervention, Information, Practitioners training, Social inequalities in health, Participatory approach

## Abstract

**Background:**

With the increase in the number of long-term survivors, interest is shifting from cancer survival to life and quality of life after cancer. These include consequences of long-term side effects of treatment, such as gonadotoxicity. Fertility preservation is becoming increasingly important in cancer management. International recommendations agree on the need to inform patients prior to treatments about the risk of fertility impairment and refer them to specialized centers to discuss fertility preservation. However, the literature reveals suboptimal access to fertility preservation on an international scale, and particularly in France, making information for patients and oncologists a potential lever for action. Our overall goal is to improve access to fertility preservation consultations for women with breast cancer through the development and evaluation of a combined intervention targeting the access and diffusion of information for these patients and brief training for oncologists.

**Methods:**

Firstly, we will improve existing information tools and create brief training content for oncologists using a qualitative, iterative, user-centred and participatory approach (objective 1). We will then use these tools in a combined intervention to conduct a stepped-wedge cluster randomized trial (objective 2) including 750 women aged 18 to 40 newly treated with chemotherapy for breast cancer at one of the 6 participating centers. As the primary outcome of the trial will be the access to fertility preservation counselling before and after using the combined intervention (brochures and brief training for oncologists), we will compare the rate of fertility preservation consultations between the usual care and intervention phases using linear regression models. Finally, we will analyse our approach using a context-sensitive implementation analysis and provide key elements for transferability to other contexts in France (objective 3).

**Discussion:**

We expect to observe an increase in access to fertility preservation consultations as a result of the combined intervention. Particular attention will be paid to the effect of this intervention on socially disadvantaged women, who are known to be at greater risk of inappropriate treatment. The user-centred design principles and participatory approaches used to optimize the acceptability, usability and feasibility of the combined intervention will likely enhance its impact, diffusion and sustainability.

**Trial registration:**

Registry: ClinicalTrials.gov. Trial registration number: NCT05989776. Date of registration: 7^th^ September 2023. URL: https://classic.clinicaltrials.gov/ct2/show/NCT05989776.

**Protocol version:**

Manuscript based on study protocol version 2.0, 21st may 2023.

**Supplementary Information:**

The online version contains supplementary material available at 10.1186/s12889-024-17719-3.

## Background

With advances in cancer treatments and the increase in long-term survivors, concerns are shifting from survival to quality of life after cancer and long-term side effects of treatments [1, 2]. Treatments combining surgery, radiotherapy, and chemotherapy are likely to alter reproductive function and quality of life after cancer [3, 4]. Access to fertility preservation is an essential element of quality of life for patients and their families after their recovery [5]. It is therefore becoming an increasingly important part of cancer management.

In many countries, recommendations include informing patients of the risks of infertility associated with cancer treatment, and referring them as soon as possible after diagnosis to a specialized center for fertility preservation consultations [6–8]. In France, since 2004, there is a legal obligation to offer fertility preservation to any woman or man exposed to a treatment likely to impair their reproductive function [9]. In 2018, a study based on National French data estimated that between 17,200 (10,400 men and 6,800 women) and 40,000 (30,000 men and 10,000 women) patients of childbearing age were eligible for fertility preservation consultations and associated fertility treatments [10]. Focusing our attention on women, fertility preservation data provided by the French National Biomedicine Agency estimated that about 750 ovarian tissue self-preservations were carried out in 2013 [11], rising to 3,500 in 2018 [12]. These figures remain very far away from the expected number of individuals concerned, highlighting the need to drastically improve information and access to fertility preservation counselling.

In Europe, a Dutch retrospective study conducted at the Radboud University Medical Center found that only 9.8% of all eligible patients under the age of 40 and seen at a university hospital in 2011 were referred for fertility preservation counselling. In addition, this study found that referral to counselling was hampered by a lack of information about the services offered by fertility preservation centers, and by a lack of collaboration among healthcare providers [13]. In Denmark, a study conducted in 2023 among young women with cancer and informed of the risks to their fertility showed that their doctors’ choice of treatment was not in favour of preserving their fertility, as survival was more important [14]. In France, a lower rate of self-reported discussion of infertility risk (46%) was reported by the 102 of 1161 oncologists who responded to a national survey between 2012 and 2013. Moreover, only 22% of oncologists reported referring the patient to a fertility center before starting treatments [15]. More recently, the FEERIC study (FErtility, prEgnancy, contRaceptIon after breast Cancer in France) showed that around 46% of survey respondents were offered specialized oncofertility counselling [16].These results are all the more worrying considering that 80% of female respondents had a high level of education, with university degrees and therefore a high level of literacy.

French regional studies carried out in Occitanie (a region in the south-west France with a population of 6 million) have shown that only 23% of women under 40 treated with chemotherapy for breast cancer received an oncofertility consultation and 8.7% received fertility preservation between 2012 et 2017. Only 44% of oncologists were confident that ovarian stimulation treatment could be used, and 29% overestimated the time required for fertility preservation [17]. Information and access to an oncofertility consultation were influenced by the type of care structure, the woman’s age, her parity at the time of diagnosis, and the metastatic status of the cancer [18]. Furthermore, another regional study showed that if all women were informed of the risks of hypo fertility related to chemotherapy, then gamete preservation could be increased by 15.35% in women aged < 30 and 22.88% in women aged 30 to 35 [19].

These data suggest that strategies are needed in France to improve access to fertility preservation. A number of teams from different countries have highlighted the importance of implementing decision-support tools to improve access to fertility preservation, particularly for younger women with cancer, but also for healthcare professionals [20]. In some French regions, information tools have been created for patients and healthcare professionals, but to our knowledge, neither these tools nor any other user-centred strategy to improve access to fertility preservation have been evaluated. Randomized trials are currently underway to evaluate the impact of these tools on the recourse to fertility preservation [21–24]. Some preliminary results are showing the importance of information for patients about the different fertility preservation options available [23, 24].

Our overall aim was to improve access to fertility preservation and related consultations for women with breast cancer through the development and evaluation of a combined intervention targeting the access and diffusion of information for these patients and brief training for oncologists.

## Methods

**Fig. 1:**
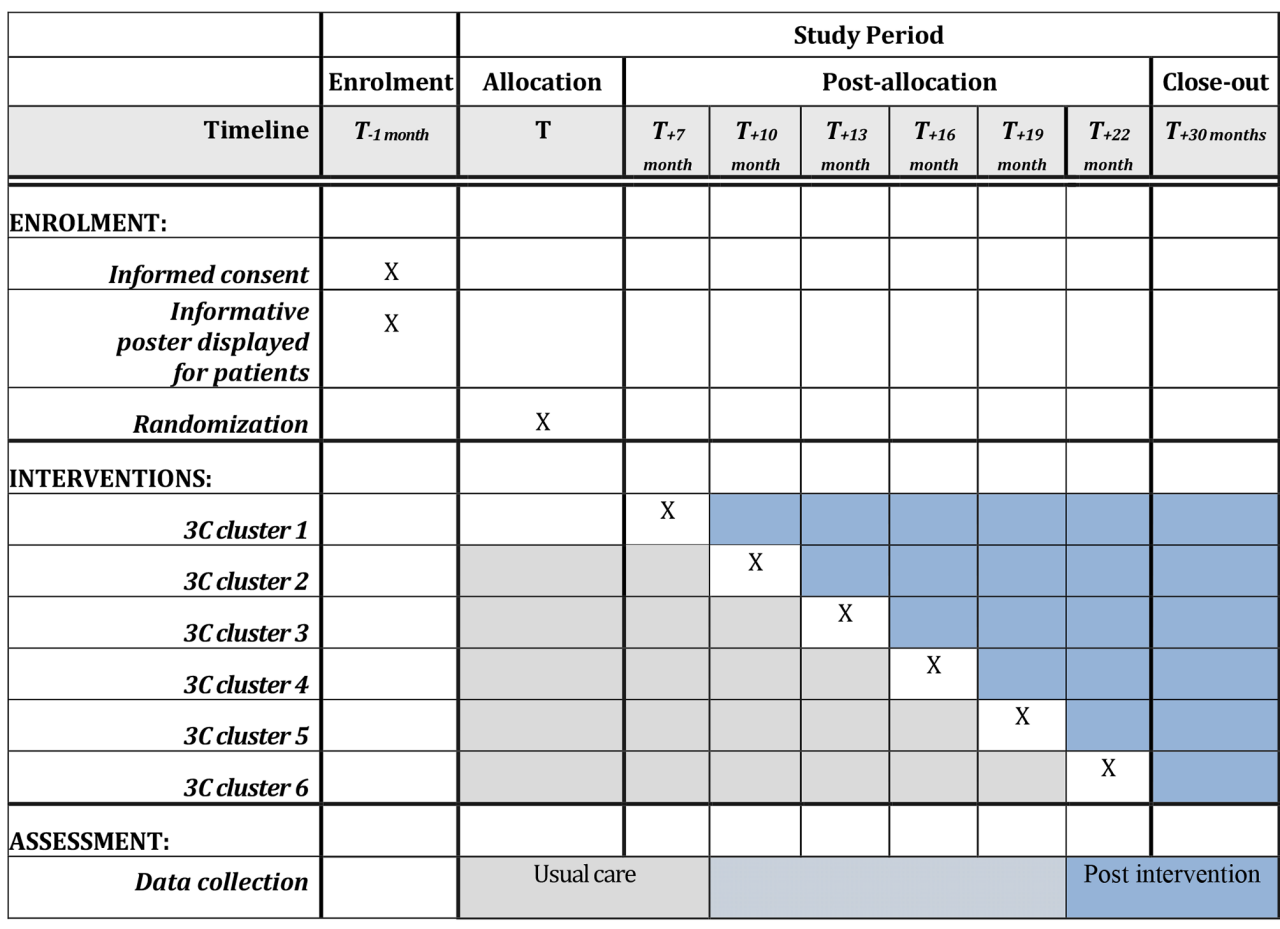
Flow diagram

The trial protocol follows the SPIRIT guidelines (see Additional File 1) and the flow diagram is presented in Fig. 1:

### Objective 1

To improve existing information tools and access about fertility preservation for patients with breast cancer and raise awareness about fertility preservation (brief training) for healthcare professionals.

#### Hypothesis 1.1

Improving access to fertility preservation services depends largely on the availability and quality of information on the risk of gonadotoxicity associated with certain cancer treatments and information on fertility preservation consultations, as well as available treatment options.

#### Hypothesis 1.2

A participatory approach, involving patients and professionals, will highlight current gaps in the information available to each stakeholder.

### Objective 2

To evaluate the impact of a combined intervention for patients and health professionals on access to fertility preservation using a stepped-wedge cluster randomized trial.

#### Hypothesis 2.1

A significant improvement in access to fertility preservation consultation (primary outcome measure) is expected after the intervention.

### Objective 3

To conduct a context-sensitive implementation analysis to identify key elements for transferability and implementation in other contexts.

#### Hypothesis 3.1

The FIC (key Function, Implementation, Context) model [25, 26] used in this approach will highlight the transferable theoretical elements that underline the activities implemented in a particular context, while taking into account the new context in which they can be implemented.

### Intervention

The planned intervention for our trial is combined, targeting both health professionals and eligible patients, it includes brochures and awareness training for healthcare professionals on fertility preservation (time required, various techniques available, prerequisites…) developed in collaboration with Regional Cancer Networks (RCNs) in Pays de la Loire et Occitanie. RCNs have been leading working groups made of oncologists, gynaecologists and biologists specialized in fertility preservation, in 2018 and 2019 respectively. The aims of these working groups were to create brochures for patients and health professionals, to facilitate interprofessional exchanges and to transmit information about fertility preservation for all cancer patients (children, men, women…). The effectiveness of the brochures created so far has not been tested, and there is no hindsight or feedback from patients or healthcare professionals regarding their acceptability, usability and usefulness.

These brochures will be adapted and improved to account for limited health literacy (HL) [27] using a participatory approach with focus groups and semi-structured interviews. Semi-structured interview guides (and focus group moderation guides) will be developed using cognitive debriefing and think-aloud techniques. We will use Morville’s user experience framework to develop all guides and questionnaires. Team members have used these techniques successfully before [28, 29]. Participants will be recruited through the RCNs. The focus groups will include patient partners, who have followed a specific training course in participatory research, and/or patients who have been affected by cancer in the past, and healthcare professionals involved in the fertility preservation process, who are members of the oncofertility working group from both participating regions. Focus groups (and interview) participants will be asked to primarily focus on reviewing the existing brochures in Occitanie and Pays de la Loire and suggesting improvements to increase acceptability, usability and adapt content for patients with limited HL. Healthcare professionals will also suggest content and format for the health professionals’ awareness-raising training. The transcripts of these focus groups and semi-structured interviews will be analysed using Nvivo 14 software [30]. We will then use the transcribed data from the focus-groups and interview to update the brochures and develop awareness-raising training. The acceptability, usability and accessibility of these updated tools will then be tested in a second round of semi-structured interviews with other patient partners and healthcare professionals.

Particular attention will be paid to the readability of the brochures. We will follow plain language recommendations [31] to improve the readability of existing brochures and address limited HL. Written contents will be checked using an online readability software (www.scolarius.com). Because this combined intervention has multiple interacting components targeting both eligible patients and healthcare professionals, it is defined as a complex intervention. We will therefore follow the updated Medical Research Council (MRC) framework for evaluating complex interventions [32].

### Trial design

**Fig. 2:**
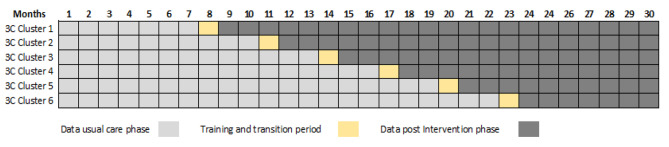
Stepped-wedge cluster randomized trial plan

We will conduct a two-arm, multi-site, stepped-wedge cluster randomized trial in two participating French regions over a 30-months period (objective 2). Centers accredited to treat cancer will be grouped into 6 clusters to define the study sites depending on geographical position. The order in which these groups will have access to the intervention will be randomized. Depending on the stage at which a group is randomized, after a period of usual care of 7, 10, 13, 16, 19, or 22 months, the intervention will run for 22, 19, 16, 13, 10, or 7 months, respectively, in each group. We chose a stepped-wedge design to examine how the effect of the intervention evolves over time, to limit the risk of contamination within a site, and to ensure that the intervention is implemented in a way that maximizes adherence to optimal use, with 1-month training and transition period (see Fig. 2). Further, this type of trial ensures the access to the intervention for all participants. Eligible patients (*N*=750) seeing participating health professionals at these sites will be recruited.

Data will be collected throughout the trial, which will allow us to compare the percentage of patients who have access to a fertility preservation consultation before and after the distribution of updated brochures and the awareness training of health professionals. This will allow us to assess the impact of these tools on the primary outcome measure.

### Setting

The study will be carried out in the Pays de la Loire and Occitanie regions, in accredited cancer centers.

### Participants

All oncologists practicing in accredited cancer centers will be invited to participate in the stepped-wedge cluster randomized trial. The managers of the RCNs involved in the project will liaise with health professionals in those cancer centers. In both regions 60 cancer licensed centers will be involved in our trial, 44 in Occitanie and 16 in Pays de la Loire. These centers will be classified in 6 randomization groups (clusters), and each cluster will have access to implemented information tools and newly created training resources for practitioners at different times as shown in Fig. 1.

#### Inclusion criteria for patients

We will include all women over 18 and under 40 years old, treated for a newly diagnosed breast cancer and receiving injectable chemotherapy in one of the participating centers.

#### Exclusion criteria for patients

There are no exclusion criteria.

### Data collection

**Fig. 3:**
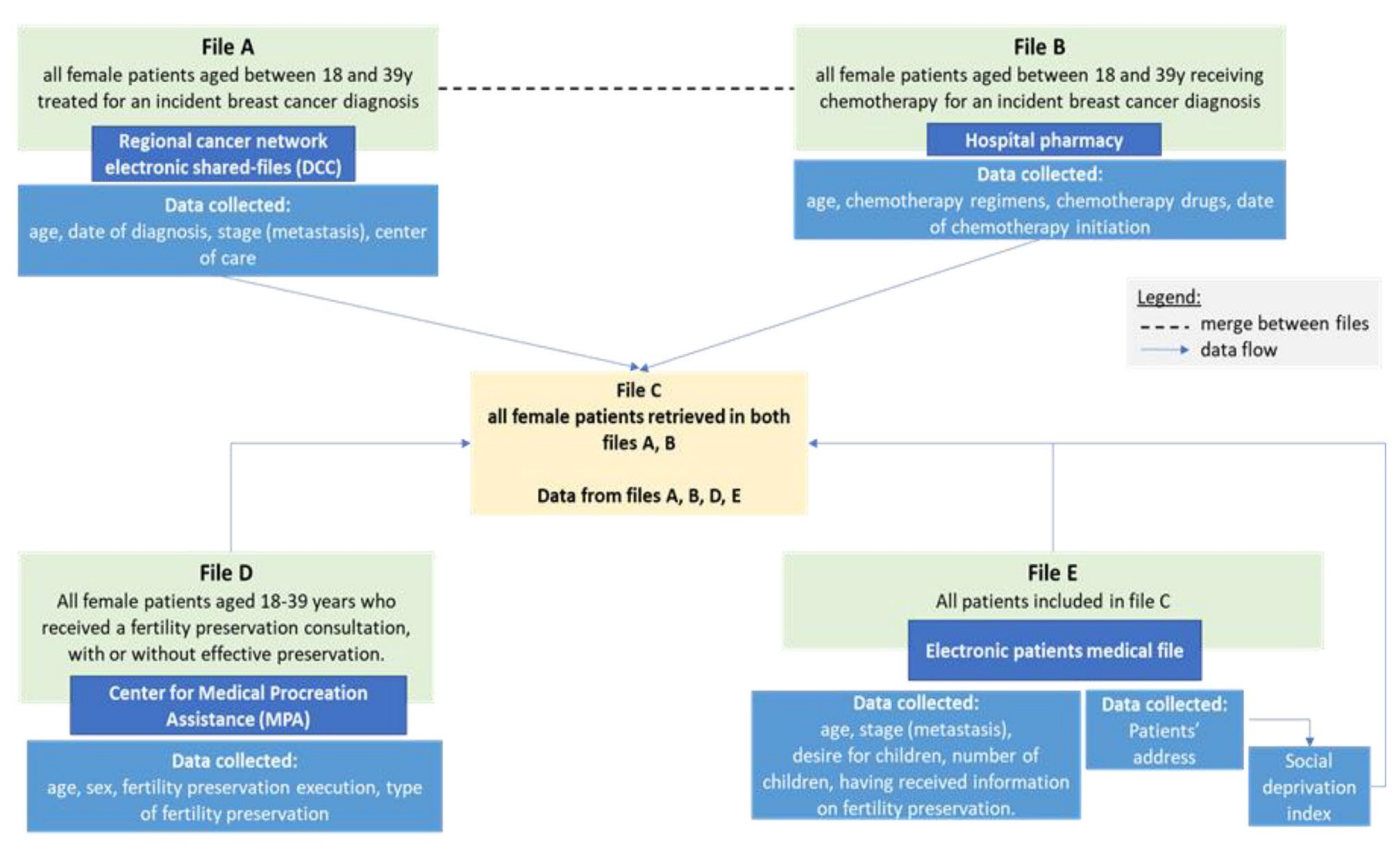
Data Architecture

As detailed in Fig. 3, we will collect patients’ age, date of diagnosis, cancer stage, and cancer center where the cancer has been diagnosed in the A-file, and type of chemotherapy start date, oncologist name, and cancer center where the patient will be treated in the B-file. Information regarding whether patients accessed a fertility preservation consultation and underwent fertility preservation will be provided by the electronic records of the assisted reproductive technology (ART) centers (File D). The number of cancers centers varies by region, requiring specific regional coordination for data collection. Data from files A, B and D can be queried directly and will not require ad hoc manual collection. On the other hand, additional data will be collected from patients’ medical records in the institutions (File E): number of children; address; presence or absence of information on fertility preservation; name of the oncologist.

### Participation and recruitment procedures for trial entry

In both regions, a Clinical Research Associate (CRA) will be responsible for collecting the data mentioned previously. This person will have access to the patients’ files and will be able to collect the necessary information. The data collected will only be stored on the servers of the RCNs that are authorized to store this type of information.

### Ethical approval, consent and recruitment strategies

The trial has received approval from the local research ethics committee (“Le Comité d’Ethique de la Recherche”) at the University of Toulouse III Paul Sabatier, France (ref 2023-609R2, dated 21st May 2023). According to French ethical regulations, as we are not modifying the care pathway and our trial is based on existing records, written or oral informed consent from patients is not required. However, following CPP (Comité de Protection des Personnes) recommendations a poster will be displayed in the health professional’s waiting room to inform patients that their personal data could be collected for our study, and the procedure for objecting will be clearly indicated. The same document will be given to patients or appended in their medical records.

### Outcome measures

#### Primary outcome measure

The primary outcome measure is the uptake rate of fertility preservation counselling for included patients.

### Secondary outcome measures

We will assess whether participants have undergone fertility preservation treatment, and which fertility treatment was chosen. Any traceable information related to the information given by doctors to patients on the fertility preservation process noted in the medical records will also be collected.

In addition, and as mentioned in the data collection section above, we will collect the following information: age, date of diagnosis, cancer stage and cancer center(s), type of chemotherapy treatment, chemotherapy start date, name of oncologist, number of children and patient’s address (for geocoding and determination of deprivation level using the European Deprivation Index). These data will be analysed in relation with the primary outcome.

### Sample size and power calculation

We will include all eligible patients over a period of 30 months starting 7 months before randomization of the first cluster and ending 7 months after randomization of the last cluster. This will allow us to reach a total sample of 750 included patients, based on a feasibility assessment of 2/3 of eligible patients recruited in the Occitanie region and 1/3 in the Pays de la Loire region. Assuming a proportion of access to fertility consultation of 23% before the intervention, with 6 clusters of about 120 patients and for an alpha risk of 5%, the smallest detectable effect of the intervention that we will be able to identify with 80% power will be 5.5% points. By knowing the date of the intervention and the date of the PCR, we will be able to identify whether patients were treated as part of usual care or in the intervention phase.

### Randomization

#### Sequence generation, type of randomization and allocation concealment

We will use an R script from the R software [33] to perform the randomization of the six participating clusters. Randomization will take place at the start of the trial, to determine the order of participation in the intervention of each of the six clusters. Eligible patients of participating health professionals in each cluster will be a priori allocated to the usual care phase before the intervention or to the intervention phase after the intervention. The random allocation sequence will be concealed to health professionals until the beginning of the intervention for their cluster (Fig. 1). The random allocation sequence will likely remain concealed to patients.

#### Changes to intervention allocation

There are no established criteria for discontinuing or modifying the allocated intervention for study participants due to the low-risk nature of the study. However, each participating health professional will be asked to record the reasons if any for patient refusal to collect data and remain in the study.

### Blinding

Due to the nature of the trial and intervention delivery, health professionals will be aware of whether they are in the usual care or intervention phase of the trial (Fig. 1). Participants will likely not be aware of which phase of the trial they are in. However, we cannot guarantee the blinding of participants throughout the trial. The data analyst will be blinded to usual care or intervention phase allocation.

### Qualitative data

The improvement of information tools and create awareness training for health professionals (objective 1), will be carried out in two phases. The first phase will consist of focus groups or semi-structured interviews to review both existing brochures previously developed by the RCNs specialist groups. Focus groups will last up to one hour, and will be conducted by videoconference separating the two categories of stakeholders: healthcare professionals and patients. We will ask focus group participants to suggest modifications and content for health professionals’ awareness training. Each focus group will include up to 12 participants. The second phase will consist of semi-structured interviews with other healthcare professionals and patients to assess the acceptability, usability and feasibility of the modified tools in routine clinical practice, integrating the results of phase 1. We will recruit 6 to 12 participants per group (one group with health professionals and one group with patient and patient partners) or until thematic data saturation is reached [34].

## Data management and statistical analysis

### Data management

The data will be archived on the SYNERGIE secure platform at the University Toulouse III (UT3) and on the EPIDEMIO server at the IUCT Oncopole of Toulouse. Health professional consent forms will also be stored digitally in a dedicated folder on the UT3 secure platform. An anonymous participant ID number will be assigned to each eligible patient of the participating health professionals. A mapping table will be established and managed by the RCN team. This mapping table will be stored digitally in a dedicated folder on a secure platform. Only the RCN project leaders will have access to it.

### Analysis plan

To identify outliers and missing data, the initial examination of the data will include descriptive statistics, frequency distributions and histograms.

#### Analyses corresponding to objective 1

**Fig. 4:**
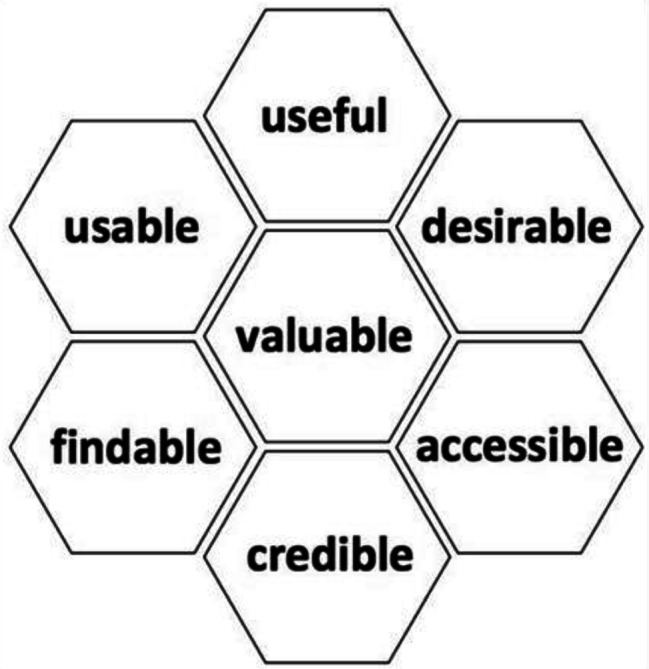
Morville’s “honeycomb” framework

We will measure the usability, acceptability, and feasibility of the complex intervention and the newly developed training content. We will use the relevant domains of Morville’s “honeycomb” framework to test the usability and acceptability of the intervention elements (see Fig. 4) [35]. We will adapt relevant elements of the complex intervention to optimize their use in routine clinical settings.

We will use Morville’s “honeycomb” framework as an analytical lens to examine the data collected in relation to our hypothesis. Observations and field notes from interviews and focus group transcriptions will also be included in the analysis. Initial descriptive codes will be generated based on the Morville frame domains. Inductive coding will also be used to capture other naturally emerging themes. Categorical codes that combine the deductive and inductive codes will be developed in a third round of coding. Dual independent coding of 20% of all transcriptions will be conducted.

#### Analyses corresponding to objective 2

In response to our overall aim, we will compare the rate of fertility preservation counselling between the usual care phase and the intervention phase. We will use linear regression models as appropriate for the primary outcome measure. Because each group is exposed to the intervention for a different length of time, the results will provide potentially valuable insights into how quickly the intervention affects the primary outcome. As we will be training several clinicians at each site, we will use a mixed-effects design in which the clinician is a random effect and the group is a fixed effect. This approach will allow to analyse pooled information from all six groups while exploiting repeated measures by clinicians in each group. Furthermore, differences between patients’ socioeconomic position will be explored. In the absence of socioeconomic data in medical files, the patient’s address will be used to assign the level of social deprivation in their area of residence, based on Townsend’s definition of deprivation as a state of social disadvantage relative to the population average [36]. We will therefore geocode the patients’ address to find the corresponding IRIS, i.e. the smallest area for which socio-economic data are available comprising nearly 2000 inhabitants. More specifically, we will use an ecological deprivation level indicator, a French version of the European Deprivation Index (EDI) [37] which has been used in many studies to characterize the socioeconomic context and approximate individual socioeconomic position, including in cancer patients [38–40].

In parallel, an explanatory study will be carried out on French Health Insurance data to assess the feasibility of estimating annually a rate of access to fertility counselling and fertility preservation as a function of the age of the patients targeted in our trial: patients over 18 and under 40 years of age treated with chemotherapy for newly diagnosed breast cancer in the two French regions mentioned previously. Based on these results, we will be able to discuss the possibility of providing adaptable and cost-effective indicators for monitoring the impact of interventions to improve access to fertility preservation.

#### Analyses corresponding to objective 3

We will use the FIC model (“key Function, Implementation, Context”) developed in a previous project in our unit [26]. Originally, it was a model for clarifying and transferring interventions aimed at addressing and reducing social inequalities in health. The FIC model, highlighted the transferable theoretical elements (key functions) that underpinned activities implemented in a particular context, while considering the new context in which they may be implemented. Because our approach will consist of describing interventions in detail to understand what worked, how, in what context, to foster sustainable implementation across regions and networks, the model will be adapted to analyse our intervention in two regions. In addition, the model will document the conditions for its transferability to other regions and other types of cancer. To do so, we will support and implement a series of workshops, bringing together the stakeholders, field actors and researchers involved in the evaluation process, in a co-constructed way. Three workshops will be organized at the time of the design and at the end of the evaluation process. They will be deployed in the two regions, thought of as two contexts for the implementation of the same intervention. Around these workshops, we will mobilize multidisciplinary researchers competent in the field and useful to the process. The workshops, in the form of focus groups, will be organized face to face or using visio conference with each team.

## Discussion

Starting with the basic assumption that a lack of information leads to a suboptimal choice, we place the quality, accessibility, readability and acceptability of information at the heart of any process requiring decision making. In the case of healthcare decisions, this seems even more important and relevant. The medical language, the dynamics of the exchanges and the often very short decision-making times do not facilitate carefully informed and deliberated upon decisions. Moreover, people’s HL is not sufficiently considered when health professionals provide medical information to them. Therefore, our work is based on an observation applicable not only to the French health system but also to foreign countries with a similar healthcare system. This highlights a lack of information and knowledge of the process of fertility preservation among patients but also among the medical profession. Due to their role as networks, the project is developed in close collaboration with the RCNs of the Occitanie and Pays de la Loire regions. Their role is essential for the dissemination of information to the different cancer centers, oncologists and other health professionals involved in the overall fertility preservation process.

Particular attention will be paid to the quality, readability (to address limited HL) and accessibility of the information provided to patients. All information materials will be written in accordance with plain language recommendations and integrate HL principles. HL will also be a key point in the training developed for health professionals. Participatory approach is already a very important point of our method. With our stepped-wedge cluster randomized trial, we hope to see a substantial difference in the percentage of people who attend at least one fertility preservation consultation. Thus, the FIC model for our trial will potentially allow us to generalize our approach to other regions of France and thus raise the crucial importance of good and clear information about fertility preservation for people with cancer.

### Electronic supplementary material

Below is the link to the electronic supplementary material.

**Supplementary Material 1**: SPIRIT checklist

## Data Availability

Data sharing is not applicable to this article as no datasets have yet been generated or analysed.

## Abbreviations

ART: Assisted Reproductive Technology
CRA: Clinical Research Associate
EDI: European Deprivation Index
FIC: Key Function, Implementation, Context
HL: Health Literacy
MPA: Medical Procreation Assistance
RCN: Regional Cancer Networks
UT3: Université Toulouse III
